# Involvement of the MetO/Msr System in Two *Acer* Species That Display Contrasting Characteristics during Germination

**DOI:** 10.3390/ijms21239197

**Published:** 2020-12-02

**Authors:** Natalia Wojciechowska, Shirin Alipour, Ewelina Stolarska, Karolina Bilska, Pascal Rey, Ewa M. Kalemba

**Affiliations:** 1 Institute of Dendrology, Polish Academy of Sciences, Parkowa 5, 62-035 Kórnik, Poland; natalia.wojciechowska@amu.edu.pl (N.W.); salipour@man.poznan.pl (S.A.); ewelina.stolarska89@gmail.com (E.S.); mgr.karolina.bilska@gmail.com (K.B.); 2 Department of General Botany, Institute of Experimental Biology, Faculty of Biology, Adam Mickiewicz University, Uniwersytetu Poznańskiego 6, 61-614 Poznań, Poland; 3 Plant Protective Proteins (PPV) Team, Centre National de la Recherche Scientifique (CNRS), Commissariat à l’Energie Atomique et aux Energies Alternatives (CEA), Biosciences and Biotechnology Institute of Aix-Marseille (BIAM), Aix Marseille University (AMU), 13108 Saint Paul-Lez-Durance, France; pascal.rey@cea.fr

**Keywords:** methionine oxidation, nicotinamide adenine dinucleotide phosphate, redox posttranslational modification, reactive oxygen species, methionine sulfoxide reductase, seeds

## Abstract

The levels of methionine sulfoxide (MetO) and the abundances of methionine sulfoxide reductases (Msrs) were reported as important for the desiccation tolerance of *Acer* seeds. To determine whether the MetO/Msrs system is related to reactive oxygen species (ROS) and involved in the regulation of germination in orthodox and recalcitrant seeds, Norway maple and sycamore were investigated. Changes in water content, MetO content, the abundance of MsrB1 and MsrB2 in relation to ROS content and the activity of reductases depending on nicotinamide adenine dinucleotides were monitored. *Acer* seeds differed in germination speed—substantially higher in sycamore—hydration dynamics, levels of hydrogen peroxide, superoxide anion radicals (O_2_^•−^) and hydroxyl radicals (•OH), which exhibited peaks at different stages of germination. The MetO level dynamically changed, particularly in sycamore embryonic axes, where it was positively correlated with the levels of O_2_^•−^ and the abundance of MsrB1 and negatively with the levels of •OH and the abundance of MsrB2. The MsrB2 abundance increased upon sycamore germination; in contrast, it markedly decreased in Norway maple. We propose that the ROS–MetO–Msr redox system, allowing balanced Met redox homeostasis, participates in the germination process in sycamore, which is characterized by a much higher speed compared to Norway maple.

## 1. Introduction

Seeds are evolutionarily important structures enabling plant reproduction. Seed germination and successful seedling establishment allow the installation of the next generation of plants. Seed germination is a complex physiological trait that can be prevented or delayed by dormancy [1,2,3]. The model of seed germination consists of three phases: seed imbibition, which is manifested by water uptake (first phase), re-initiation of metabolic processes (second phase) and postgermination growth (third phase), which refers to a further increase in water uptake that results in embryo expansion [1]. Germination sensu stricto involves first and second phases [1,2,3]. The molecular basis of the changes in morphology, structure, metabolism, hormones and gene expression during seed germination is well characterized in *Arabidopsis* [4,5]. Among phytohormones, abscisic acid (ABA), giberrellins (GAs) and ethylene (ET) primarily regulate seed dormancy and germination in dicot species [6,7], notably through interplay with reactive oxygen species (ROS) [8,9]. Many studies confirmed that hormonal regulation of germination has effects at the transcriptional and proteome levels [10,11] and eventually results in global changes in enzyme activity [12]. Global interactions exist also at the transcriptome level in germinating seeds [13]. Dry mature seeds can accumulate over 10,000 mRNAs intended for the synthesis of proteins associated with redox regulation, glycolysis and protein metabolism [14]. Importantly, the activity of many enzymes is redox-regulated [12], and their oxidized forms in mature dry seeds are reduced during imbibition for metabolism restoration [15]. In this context, redox posttranslational control of seed germination has emerged as an extremely important process [14,16].

ROS are assumed to be signaling molecules, and the interplay between ROS-related transduction pathways and others, such as hormone-related pathways, is well described [17,18,19,20,21]. Among ROS, the signaling role of hydrogen peroxide (H_2_O_2_) is widely studied, particularly in plant acclimation to stress and during growth and development [22,23,24]. ROS are signaling molecules in seeds that modulate dormancy disruption and germination [25,26] and are involved in endosperm deterioration and seed reserve mobilization [27]. Three ROS, H_2_O_2_, superoxide anion radicals (O_2_^•−^) and hydroxyl radicals (•OH), have been implicated in germination, particularly in root development [28,29,30]. H_2_O_2_ and O_2_^•−^ were demonstrated to be associated with cell differentiation and proliferation, respectively [30]. Postgerminative axis growth involves O_2_^•−^ production [31,32,33]. Less attention has been paid to the role of the short-lived but most reactive ROS, •OH [34]. Indeed, •OH is involved in germination [35,36,37]. More and more evidences indicate that •OH could be targeted to play a role in plant cell wall loosening thus placing these reactive molecules as important components involved in plenty of developmental processes [38]. Miller [39] and Fry [38] showed that cell-wall polysaccharides such as pectin and xyloglucan can be broken down by •OH. Further analyses confirmed that •OH production in the apoplast causes scission of specific cell wall polysaccharides in elongating maize coleoptiles as well as in the radicles and endosperm caps of germinating cress seeds [35]. 

ROS are thus signaling molecules involved in the regulation of seed germination [25]. Nucleic acids, especially RNA, and proteins are the most sensitive molecules to oxidation [40]. Targeted mRNA oxidation can fine-tune the cell signaling pathway that controls germination via selective translation [41]. Massive protein oxidation occurs during germination [42,43]. Importantly, selective oxidation of both mRNA and proteins is necessary for completing germination [25,27,41,44]. ROS damage to proteins can be irreversible and irreparable (i.e., carbonylation) [45]. Reversible protein oxidative modifications involve cysteine (Cys) and methionine (Met), which are the two amino acids the most prone to oxidation by ROS [46]. Oxidation of Met to methionine sulfoxide (MetO) is reversed via the action of enzymes termed methionine sulfoxide reductases (Msrs), including several isoforms that are classified as one of two types, A and B [36,37,47,48,49,50,51]. The Met-MetO-Met transition in proteins is considered a redox switch-regulating activity [48] in relation to ROS-initiated signaling. The reversibility of the Met redox status depends on the activity of the Msrs system, which in turn depends on the presence of redoxins and reducing agents that can regenerate Msrs [37,52]. Met metabolism was demonstrated to be essential to seed germination [16]. More precisely, proteins associated with Met synthesis and the recycling pathway [10] were identified as important for dormancy disruption [53]. However, Met redox homeostasis has never been investigated in this context.

Norway maple (*Acer platanoides* L.) and sycamore (*Acer pseudoplatanus* L.) belong to the same genus but produce desiccation-tolerant and desiccation-sensitive seeds, respectively. The comparison of Norway maple and sycamore seeds became a model for studying differences between orthodox and recalcitrant seeds at important transitions, such as development [54,55], dormancy acquisition [53,56] and drying/desiccation [57,58,59,60,61]. However, the germination process was studied uniquely in the two species in the context of nuclear replication activity [62] and hormonal regulation [11]. 

The seeds of these two contrasting *Acer* species were characterized by distinct levels of ROS, MetO, MsrB1 and MsrB2 and differences in the activity of NADPH-dependent reductases during the development and maturation phases [63] as well as during dehydration and desiccation [64]. The MetO/Msr system was assumed to be involved in the establishment of desiccation tolerance in orthodox Norway maple seeds [64]. Thus, it was necessary to determine whether the MetO/Msr system participates in the regulation of germination, during which Norway maple and sycamore seeds cease to be different in terms of desiccation tolerance [1,65]. Of note, accumulation of MsrB was reported to be related to re-establishment of desiccation tolerance in germinating seeds [66], suggesting that MsrBs might contribute to proper germination. Therefore, we investigated whether these contrasted *Acer* seeds also exhibit distinct features in terms of ROS levels, MetO content, MsrB abundance and the activity of NAD(P)H-dependent reductases in relation with their germination characteristics.

## 2. Results

### 2.1. Germination Patterns and Water Status

**Figure 1:**
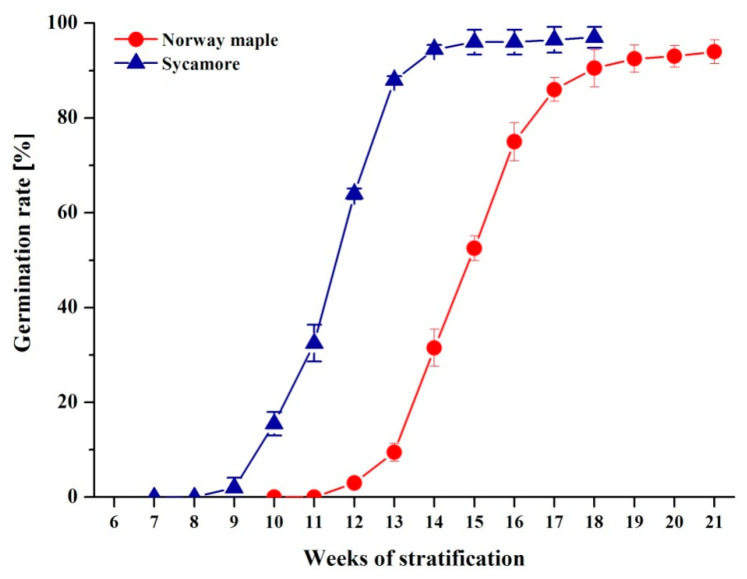
Germination curves representing Norway maple and sycamore seeds subjected to germination. Data are the means of four independent replicates ± the standard deviation.

We first investigated the germination characteristics in Norway maple and sycamore seeds (Figure 1). Norway maple embryonic axes started to protrude at the 12th week after imbibition (WAI), and over 5 weeks, over 90% of seeds passed into the postgerminative growth phase. The germination capacity of the Norway maple seedlot reached 95 ± 2.3%. The dynamics of germination were strikingly different in sycamore seeds. Radicle protrusion began earlier at the 9–10th week, and three weeks later, over 90% of seeds exhibited visible radicles. The final germination capacity of sycamore seeds was high and reached 97 ± 2.2%. In this context, both types of *Acer* seeds were highly viable; however, Norway maple required a longer time (3 weeks) to initiate the protrusion of radicles. The germination speed index (GSI) confirmed that sycamore seeds accomplished the germination process faster (GSI = 6.72 ± 0.16) than seeds of Norway maple (GSI = 4.41 ± 0.14).

**Figure 2:**
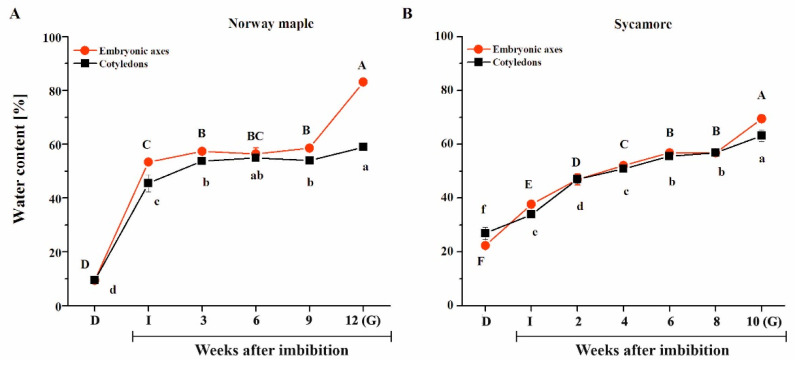
Water sorption isoterms displaying the hydration of the embryonic axes and cotyledons of Norway maple (**A**) and sycamore (**B**) dry seeds and during germination. Abbreviations: D, Dry seeds; I, Imbibed seeds; G, Germinated seeds. Data are the means of three independent replicates ± the standard deviation. Identical letters indicate groups not significantly differentiated according to Tukey’s test.

During germination, the water status of Norway maple seeds exhibited a pattern similar to the typical scheme observed in many species (Figure 2A). Imbibition caused a spectacular increase in water content (WC), particularly in Norway embryonic axes, whereas the WC of cotyledons was 5–10% lower. Then, the WC remained relatively stable throughout nine WAI. A subsequent peak in tissue hydration was reported in embryonic axes at the 12th WAI, while germination was manifested by radicle protrusion, and an almost 5% WC increase was reported in cotyledons. Sycamore seeds absorbed water in a very distinct manner (Figure 2B). The increase in WC was more linear and depended on the germination phase duration (R^2^ = 0.86). Sycamore embryonic axes definitely achieved a higher WC at the 10th WAI, while the elongation of radicles appeared. To summarize, Norway maple and sycamore seeds exhibited entirely different hydration patterns during germination. Remarkably, the sycamore seeds, which displayed fast germination compared to Norway maple seeds, were also characterized by a very progressive increase in water content.

### 2.2. ROS Levels in Germinating Acer Seeds

**Figure 3:**
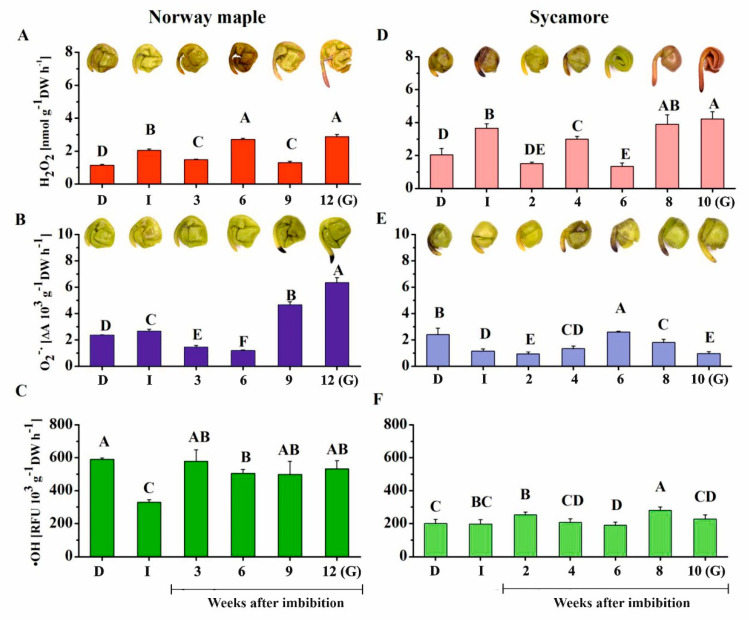
Measurements of ROS release levels. (**A**) Hydrogen peroxide (H_2_O_2)_, (**B**) superoxide anion radicals (O_2_^•−^) and (**C**) hydroxyl radicals (•OH) in dry and germinating Norway maple (**A**–**C**) and sycamore (**D**–**F**) seeds as revealed by histochemical detection of H_2_O_2_ and O_2_^•−^, which were visualized as brown staining and dark blue staining, respectively. Abbreviations: D, Dry seeds; I, Imbibed seeds; G, Germinated seeds. Data are the means of six independent replicates ± the standard deviation. Identical letters indicate groups without significant differences according to Tukey’s test.

ROS release levels were assayed in *Acer* seeds during germination, as ROS are well recognized effectors in redox signaling during this developmental stage [25,26,27]. Further, they also modulate protein redox status, particularly in Cys and Met residues [46]. In Norway maple seeds, imbibition resulted in the doubling of H_2_O_2_ release; however, at the 3rd WAI, a decrease was recorded (Figure 3A). Three weeks later, H_2_O_2_ release doubled and then decreased at the 9th WAI and peaked again in germinating seeds, where the highest level was recorded. Imbibition caused an increase in O_2_^•−^ levels in Norway maple seeds (Figure 3B). After that, O_2_^•−^ levels decreased up to the 6th WAI. Three weeks later, the level strongly increased, reaching three- and five-fold higher levels than that in dry seeds and in seeds at the 6th WAI, respectively. Interestingly, imbibition resulted in a 50% decrease in release of •OH (Figure 3C). Then, •OH levels increased and remained stable up to germination.

Similarly to Norway maple, imbibition of sycamore seeds was associated with an increase in the H_2_O_2_ release level (Figure 3D). Then, this level declined, increased at the 4th WAI and declined again two weeks later. The highest levels of H_2_O_2_ were detected at the two final stages and were twice as high as those in dry seeds. In contrast to Norway maple seeds, imbibition halved the levels of released O_2_^•−^ in sycamore seeds (Figure 3E). A peak in O_2_^•−^ release was measured at the 6th WAI, after which O_2_^•−^ levels progressively decreased. No great change was observed regarding •OH content in sycamore except the somewhat higher levels at the 2nd and 8th WAI (Figure 3F).

Histochemical detection of hydrogen peroxide and superoxide anion radicals was performed to determine the seed parts, in which ROS present. ROS were detected in embryonic axes and cotyledons of both species (Figure 3). Intense staining revealing the presence of H_2_O_2_ was detected at the 6th WAI in Norway maple seeds, whereas a strong signal of O_2_^•−^ was reported at the tip of embryonic axes at 9–12th WAI. Sycamore seeds exhibited more intense H_2_O_2_ staining, particularly at the germinating stage. Most interestingly, all these observations corresponded to biochemical measurements. To conclude, similar dynamics in the changes in H_2_O_2_ levels were reported in both types of *Acer* seeds, with higher concentrations in sycamore, whereas very different patterns were observed for the two other ROS, which were less abundant in sycamore, particularly O_2_^•−^.

### 2.3. MetO Content in Germinating Acer Seeds

**Figure 4:**
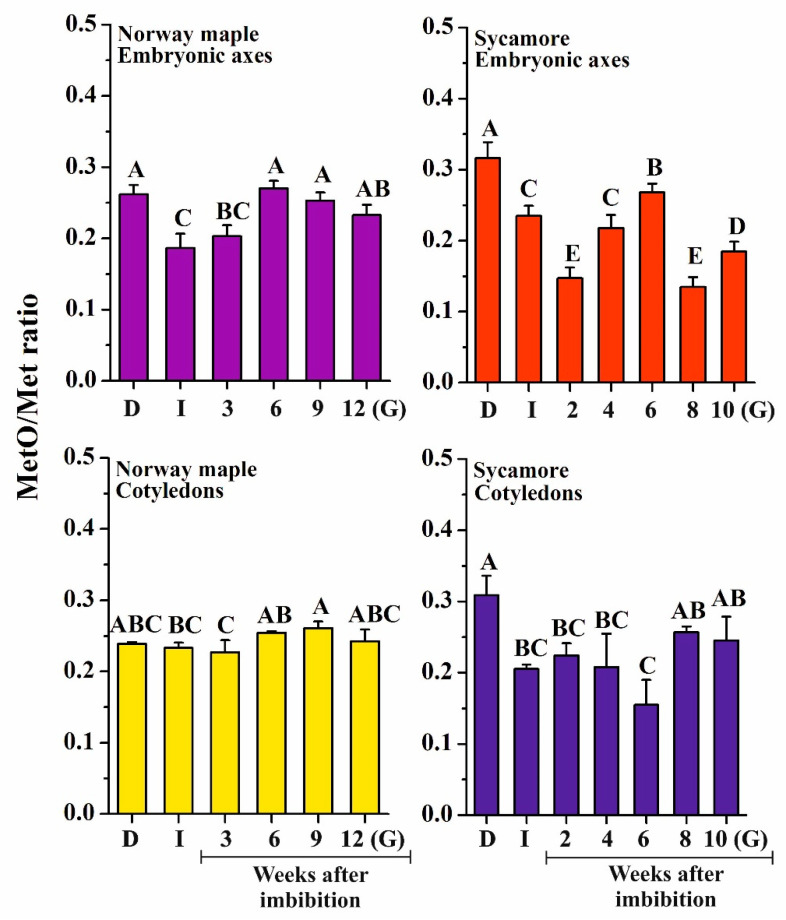
Measurement of protein-bound methionine sulfoxide (MetO) levels in dry and germinating Norway maple and sycamore seeds. Abbreviations: D, Dry seeds; I, Imbibed seeds; G, Germinated seeds. Data are the means of three independent replicates ± the standard deviation. Identical letters indicate groups not significantly differentiated according to Tukey’s test.

ROS induce oxidation of proteins, particularly at the level of sulfur-containing amino acids, such as Met. Peptide-bound MetO levels were measured to determine whether they follow the changes in ROS levels. Initially, sycamore dry seeds displayed higher MetO content than Norway maple desiccated ones (31% and 26%, respectively) (Figure 4). Interestingly, at the end of the germination process, seeds of both species contained similar MetO content; however, the dynamics of MetO changes during germination stages were entirely different. Except in Norway maple cotyledons, imbibition resulted in a significant decrease in MetO levels, and low levels were maintained for the first two weeks after imbibition. Remarkably, the sycamore embryonic axes exhibited much lower MetO contents (~14%) at 2nd and 8th WAI. Of note, the embryonic axes of both *Acer* species displayed a peak in the MetO level at the 6th WAI. In conclusion, our data revealed much more pronounced variations in MetO level in sycamore compared to those in Norway maple, particularly in embryonic axes.

### 2.4. MsrB1 and MsrB2 Abundance in Germinating Acer Seeds

**Figure 5:**
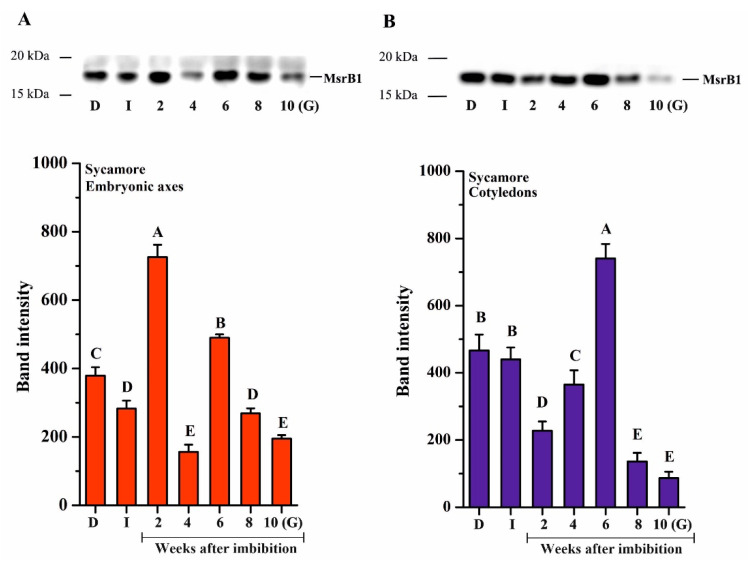
Western blot analysis and densitometry analysis of methionine sulfoxide reductase (Msr)B1 protein in the embryonic axes (**A**) and cotyledons (**B**) of dry and germinating sycamore seeds. Abbreviations: D, Dry seeds; I, Imbibed seeds; G, Germinated seeds. The data are the means of three independent replicates ± the STDs. The same letters indicate groups that were not significantly different according to Tukey’s test.

**Figure 6:**
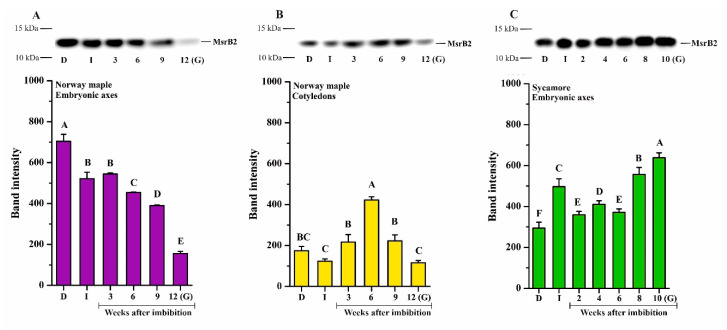
Western blot analysis and densitometry analysis of MsrB2 protein in the embryonic axes (**A**) and cotyledons (**B**) of dry and germinating Norway maple seeds and in the embryonic axes of D and germinating sycamore seeds (**C**). Abbreviations: D, Dry seeds; I, Imbibed seeds; G, Germinated seeds. The data are the means of three independent replicates ± the STDs. The same letters indicate groups that are not significantly different according to Tukey’s test.

Oxidation of Met to MetO is reversible thanks to the action of Msr enzymes. Two isoforms related to type-B, B1 and B2, have been previously reported to fulfill essential roles in seed development or longevity [64,67]. We thus investigated the abundance of these two enzymes using Western blotting analysis during the germination process in *Acer* seeds (Figure 5 and Figure 6). In contrast to Norway maple, both isoforms were detected in sycamore. In the embryonic axes, MsrB1 was detected in all of the studied stages, with a varying abundance (Figure 5). Imbibed sycamore displayed decreased MsrB1 content; a strong increase was noted at the 2nd and 6th WAI, and the protein abundance decreased significantly during the subsequent weeks. A relatively low protein level was detected in germinated seeds (Figure 5A). In cotyledons, the level of MsrB1 in dry and imbibed seeds was similar. From the 2nd to the 6th WAI, a gradual increase in MsrB1 protein level was detected, with a substantial peak at week 6. At week 8, the amount of protein dropped sharply, and it was almost undetectable in germinated seeds (Figure 5B).

The MsrB2 protein was detected in embryonic axes and cotyledons of Norway maple (Figure 6A,B) and in the embryonic axes of sycamore (Figure 6C). A relatively high protein amount was detected in dry Norway maple embryonic axes (Figure 6A). Following imbibition, the level of MsrB2 was somewhat reduced and further gradually decreased in the subsequent weeks. At the 9th WAI, the protein abundance was very low, whereas in germinated seeds, it was barely detectable (Figure 6A). In cotyledons, the amount of MsrB2 detected in all studied stages was lower compared to that in embryonic axes (Figure 6B). There was a slight decrease in protein content following imbibition. In the subsequent weeks, the MsrB2 abundance was higher, particularly at the 6th WAI, while in germinated seeds, the protein level was significantly reduced (Figure 6B). In germinated Norway maple seeds, the MsrB2 protein was almost undetectable.

In sycamore embryonic axes, a relatively high level of MsrB2 abundance was detected at all studied stages (Figure 6C). In imbibed seeds, the protein amount was higher than that in dry seeds. In the last weeks of germination sensu stricto phase, the protein amount further increased, reaching the highest level in germinated seeds. Interestingly, MsrB2 displayed reverse patterns of abundance in Norway maple and sycamore seeds during germination. A gradual increase in MsrB2 abundance was detected in sycamore, whereas a gradual decrease was reported in Norway maple.

### 2.5. Activity of NAD(P)H-Dependent Reductases in Germinating Acer Seeds

Oxidoreductases constitute the major class of enzymes that catalyze a wide variety of redox reactions using different substrates. This class includes NADH- and NADPH-dependent groups of enzymes, with a relatively small group of oxidoreductases using both cofactors [68]. The activity of NAD(P)H-dependent reductases reflects the global cell redox status and the ability to maintain redox homeostasis via effectors such as Msrs. Importantly, the regeneration system for cytosolic and some plastidial Msrs is based on NADPH-dependent mechanisms [69]. In other respects, the source of the reducing power of plastidial Msrs, such as MsrB2, in a nonphotosynthetic context like seeds remains unknown.

**Figure 7:**
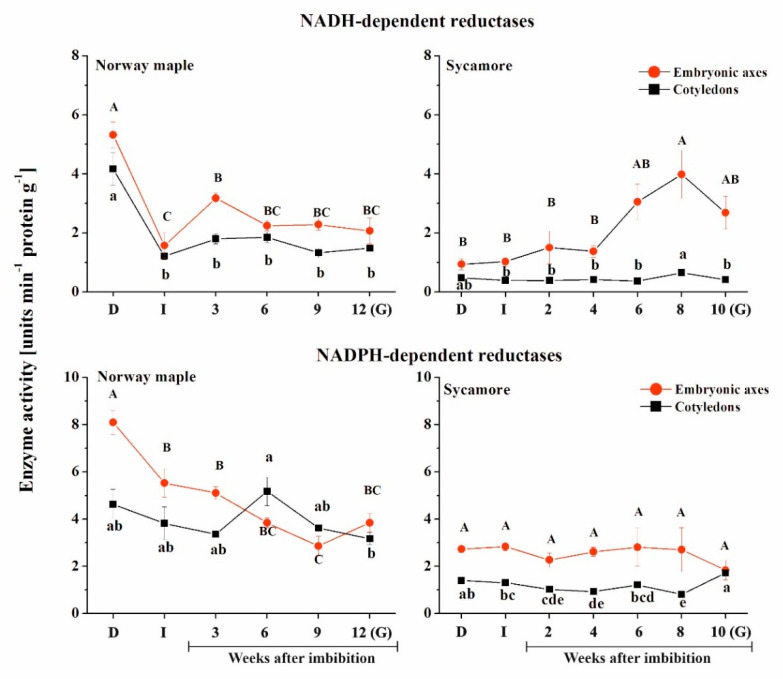
Activity of NAD(P)H-dependent reductases in the embryonic axes and cotyledons of Norway maple and sycamore dry and germinating seeds. Abbreviations: D, Dry seeds; I, Imbibed seeds; G, Germinated seeds. Statistically significant differences are indicated with different letters (one-way ANOVA, followed by Tukey’s test at *p* ≤ 0.05).

When comparing both *Acer* species, the greatest difference in the activity of NADH-dependent reductases was reported in dry seeds (Figure 7). At this stage, a much higher activity level was measured for NADH-dependent reductases in Norway maple seeds compared to sycamore. Then, a strong decrease (−66%) was observed upon imbibition in both cotyledons and embryonic axes of Norway maple. It is important to note that the peaks of activity were detected at distinct stages of the seed germination process in the two species. In Norway maple embryonic axes, the activity of NADH-dependent reductases peaked at the 3rd week. Then, relatively constant and similar activity was sustained up to the germinated stage as well as in cotyledons. In contrast, in embryonic axes of sycamore, the activity of NADH-dependent reductases was quite similar and low up to the 4th WAI and then substantially increased and peaked at the 8th week, with a 4-fold higher level compared to that of the initial stage. No variation was detected in sycamore cotyledons, the activity being quite low throughout the germination process. Interestingly, the activity of NADH-dependent reductases was twice as low in sycamore cotyledons compared to that in Norway maple cotyledons.

The activity of NADPH-dependent reductases was higher in Norway maple compared to sycamore (Figure 7). In embryonic axes of Norway maple, a peak was detected in dry seeds, the activity being twice as high as that in cotyledons at this stage. Then, the activity gradually decreased in axes, while in cotyledons, a peak was observed at the 6th WAI. Sycamore seeds displayed lower activity of NAD(P)H-dependent reductases compared to Norway maple. The activity was constant in embryonic axes throughout the germination stages. Sycamore cotyledons exhibited activity that was twice as low as Norway maple activity, except in germinated seeds, where a similar level was measured in both embryonic axes and cotyledons of this species.

### 2.6. Analysis of Correlations between Studied Parameters

**Figure 8:**
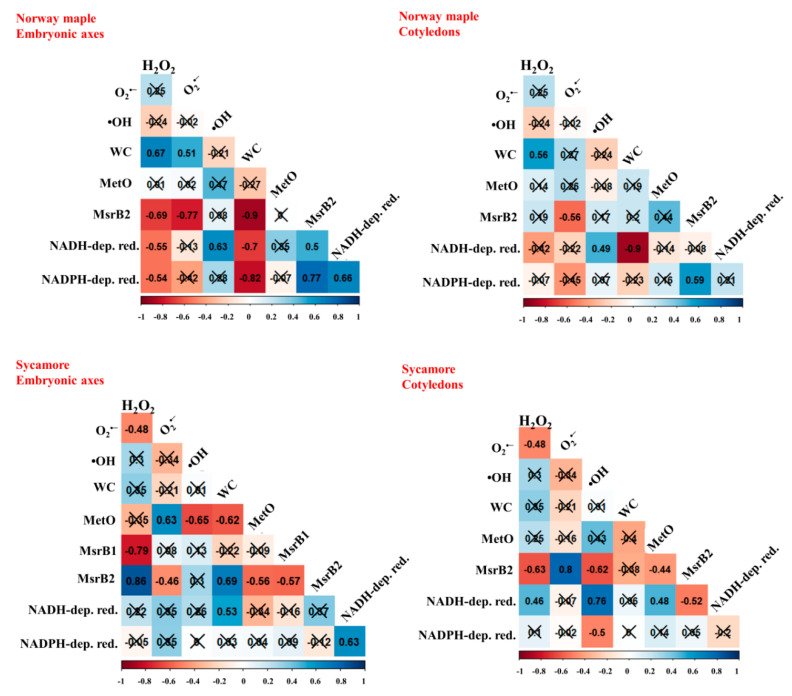
Correlation matrices calculated for embryonic axes and cotyledons of Norway maple and sycamore dry and germinating seeds based on levels of hydrogen peroxide (H_2_O_2_), superoxide anion radical (O_2_^•−^) and hydroxyl radical (•OH), water content (WC), levels of protein-bound methionine sulfoxide (MetO), the abundance of methionine sulfoxide reductase isoforms (MsrB1, MsrB2), activity of NADH-dependent reductases (NADH-dep. red.), activity of NADPH-dependent reductases (NADPH-dep. red.). The percentage of WC was ArcSin transformed. Crossed numbers indicate non-significant correlation (*p* > 0.05). Th more red color the more negative correlation, whereas the more blue the more positive correlation.

To investigate the relationship between the levels of ROS, MetO and Msrs during germination in orthodox and recalcitrant *Acer* seeds, Pearson correlation analyses were made between all the parameters tested (Figure 8). The MetO content was correlated with the levels of ROS only in sycamore seeds and was positively correlated with O_2_^•−^ release values and negatively with •OH ones. Furthermore, in this species, the MetO content was negatively correlated with the MsrB2 abundance in embryonic axes and positively correlated with the MsrB1 abundance in cotyledons. The abundance of MsrB1 was positively correlated with the activity of NADH-dependent reductases. In Norway maple, no significant relationship between ROS levels, MetO content and Msrs abundance could be detected. However, the MsrB2 abundance was positively correlated with the activities of NADH- and NADPH-dependent reductases in embryonic axes and of NADPH-dependent reductases in cotyledons. 

## 3. Discussion

Seeds of Norway maple and sycamore were reported to differ in the levels of ROS, MetO, the abundance of MsrB1 and MsrB2, and the activity of NADPH-dependent reductases during maturation and drying [63,64]. The control of Met redox homeostasis by the MetO/Msr/Met system was proposed to accompany the acquisition of desiccation tolerance in Norway maple seeds [57,64]. As the role of the MetO/Msr/Met system has not yet been documented in seeds during germination, we investigated whether this system could be involved in the regulation of germination in the two *Acer* species because desiccation tolerance is lost in orthodox seeds during this developmental stage [1] and more precisely coincides with visible radicle elongation [70,71]. Thus, searching for possible regulatory functions of the MetO/Msrs system seemed very interesting, particularly taking in consideration the important role of Met synthesis and recycling in seed germination [10,16].

### 3.1. Differential Behavior of Acer Seeds during Germination

Under identical stratification conditions, sycamore seeds exhibited a higher GSI and germinated faster than Norway maple seeds (Figure 1). The time needed for the achievement of germination phase III manifested by radicle protrusion is different in *Acer* species and takes 12–20 weeks for Norway maple and 8–15 weeks for sycamore [72]. Consistently, we observed that sycamore seeds began to elongate their radicles three weeks earlier than Norway maple seeds (Figure 1). Cold storage was reported to eliminate dormancy in *Acer* and accelerate germination of Norway maple seeds [11], but here, the analyzed seeds were not stored and directly subjected to germination after harvesting. This 3-week difference could be related to the discrete hormonal balance since mature sycamore seeds contain less ABA than Norway maple seeds [73], as reflected by the degree of the physiological dormancy of the two species. A dynamic balance of two antagonistic hormones, ABA and GAs, controls seed dormancy and germination [6,7]. Consequently, ABA decreased, while GA increased the germination rate but not the germination speed in Norway maple seeds [11]. In contrast, GA substantially increased the germination rate and germination speed in sycamore seeds [53]. In particular, ABA and GA levels were reported to substantially affect the proteome of germinating Norway maple seeds [11] and sycamore seeds [53]. Additionally, the hormone balance supported by ROS homeostasis enables successive germination [74]. Particularly in imbibed seeds, H_2_O_2_ increases the level of ABA [8] and induces synthesis of ethylene [75]. Thus, ROS signals combined with GA- and ABA-related signaling transduction pathways fulfill key roles in the germination process that need to be further delineated. The progressive increase in water content observed in sycamore seeds (Figure 2), which does not fit the classical triphasic seed germination model [1], might be as well related to differential behavior of *Acer* seeds during germination.

### 3.2. Relationship between ROS and MetO Levels during Germination of Sycamore Seeds

ROS, including H_2_O_2_, O_2_^•−^ and •OH, participate in regulating the germination process in seeds, particularly root development [25,29,30,74,76]. Bailly et al. [70] established the concept of the “oxidative window for germination” linked to a specific range of ROS levels, which leads to the initiation and further progression of seed germination. Germinated Norway maple and sycamore seeds contained 3 nM and 4 nM g^−1^ DW of H_2_O_2_ (Figure 3). Importantly, H_2_O_2_ concentrations ranging from 1–10 nM are assumed to be physiological concentrations involved in signaling [77]. Thus, H_2_O_2_ levels, which are slightly higher in sycamore seeds, are within the signaling range, indicating that the peaks in ROS might result in the high germination rate recorded in both species (Figure 1). 

H_2_O_2_ is the major ROS type that is considered a signaling molecule during seed germination [25]. H_2_O_2_ provides the optimum oxygen concentration for faster imbibition and germination [78]. The peak of H_2_O_2_ detected at the 4th and 6th WAIs in sycamore and Norway maple seeds, respectively, possibly reflects the signaling function in the early stages of dormancy disruption. This two-week delay is very likely associated with the different dynamics of germination because the time needed for initiation of radicle protrusion is longer in Norway maple [72] and lasted three weeks in our studies (Figure 1). Higher H_2_O_2_ levels together with the occurrence of peaks at earlier stages of the germination sensu stricto phase are likely to contribute to the faster germination of sycamore seeds. As a result of faster water uptake, earlier peaks of ROS were the indices of rapid germination rate of recalcitrant *Avicennia marina* seeds [79]. Evidently, Norway maple and sycamore display different germination-related ROS signaling patterns. A clear increase was reported in the concentrations of O_2_^•−^ in Norway maple seeds at the final germination stages, which is in line with the fact that postgerminative axis growth requires O_2_^•−^ [31,32,33]. Interestingly, the highest •OH levels appeared just before radicle elongation uniquely in sycamore seeds. •OH functions in cell wall loosening [34]; hence, the burst of •OH reported during radish germination [80] and in our studies (Figure 3) can be assumed to be an important component of the germination program.

Among ROS, •OH has the highest oxidative potential in Met oxidation [48], and a correlation between the levels of •OH and MetO was observed in sycamore seeds during germination (Figure 8). Interestingly, •OH was shown to affect MetO levels in desiccated Norway maple embryonic axes [64]. Another ROS type causing Met to oxidize is H_2_O_2_ [48]. Mature Norway maple and sycamore seeds differed in MetO content and showed distinct responses to dehydration and desiccation [64], with no change in sycamore and a decrease in the MetO level in Norway maple along with desiccation. In contrast, substantial changes in the MetO content were observed upon germination, more specifically in sycamore seeds (Figure 4). Sycamore embryonic axes displayed further decreased MetO levels at the 2nd WAI (Figure 4) and a peak at the 6th WAI coinciding with that of O_2_^•−^ (Figure 3), as reflected by the positive correlation between the two parameters. In plants, the MetO content differs substantially depending on physiological context, organ type and very likely ROS homeostasis and scavenging capacity. For example, unstressed leaves from various species contain 2–6% MetO, and environmental constraints were reported to increase this value up to more than 50% [81,82,83]. The oxidation state characteristic of seeds [27] was particularly reflected by the high MetO level exceeding 30% in dried *Acer* seeds [64]. The dynamically changing MetO level in sycamore germinating seeds (Figure 4) is consistent with the fact that controlled protein oxidation is required for successive seed germination [27]. The fine control of Met redox status in concert with ROS signals and Msr activity very likely modulates protein functions through posttranslational modification [84]. 

### 3.3. Involvement of MsrB2 in Sycamore Seed Germination

MetO is easily reduced by Msr enzymes present in all living organisms [85]. It is supposed that in plants, Msr enzymes play more complex roles than in other organisms such as yeast or mammals because of the much larger number of isoforms in subcellular compartments [37,86]. In this work, we focused on the two isoforms of plastidial MsrB proteins, namely, MsrB1 and MsrB2. Initially, the functions of these proteins were considered only in photosynthetic tissues [20,36]. Currently, their presence has also been confirmed in other organs such as flowers, stems, roots and seeds [67,87,88]. Most available information about their functions relates to their contribution in responses to biotic [89] and abiotic stresses [87,90]. However, these isoforms are also involved in several developmental processes such as seed maturation, desiccation and longevity [63,64].

In sycamore seeds, both MsrB isoforms were detected, notably with an increasing level of MsrB2 in embryonic axes during germination sensu stricto phase (Figure 6). In Norway maple seeds, only one isoform, MsrB2, was detected using western blot analysis as reported during seed maturation [63] and desiccation [64]. The low abundance of MsrB1 in Norway maple seeds might be compensated by MsrB2 or other Msrs including MsrA as reported by Staszak and Pawłowski [56]. The high MsrB2 abundance in sycamore seeds very likely results in enhanced Msr activity and thus reduction of oxidized proteins that could delay germination in their oxidized state [91]. At the present time, very little is known regarding targets of Msrs in plants. In Arabidopsis leaves, among MsrB1 partners, 13 plastidial and 11 nonplastidial proteins were identified [92]. They included elongation factor 2 and 26S proteasome regulatory subunit, which are very sensitive to oxidation [93,94]. Therefore, protein synthesis and turnover can be processed more efficiently, further contributing to germination speed, particularly in sycamore seeds due to the high abundance of MsrB2 in this species. Interestingly, glyceraldehyde-3-phosphate dehydrogenase (GAPDH) was identified as an MsrB1 partner [92]. Reduced GADPH acts in glycolysis releasing NADH, whereas oxidized GADPH redirects glycolysis to the pentose phosphate pathway (PPP), allowing the cell to generate more NADPH [95]. Thus, more targets of Msrs need to be identified, particularly in seeds, to unravel the roles of these reductases during germination. Most interestingly, MsrB2 uniquely exhibited a linear increase in abundance in embryonic axes of germinating sycamore seeds (Figure 6), likely indicating upregulation of *MsrB2* gene expression in this species. The promoter regions of both *MsrB1* and *MsrB2* contain several ABA responsive elements, GA and ABA responsive elements and binding sites for GA-regulated transcription factors [96]. In this context, plant hormones, predominantly ABA and GA, which are involved in regulation of MsrBs, might be associated with control of the MetO/Msr system in germinating *Acer* seeds. Increasing MsrB2 abundance in sycamore embryonic axes (Figure 6) seems to have a beneficial effect on significantly reduced MetO content during all germination stages (Figure 4). Recently, methionine sulfoxidation of a nonripening transcription factor was identified as a posttranslational modification involved in regulation of tomato ripening [97]. Thus, the support of MsrBs in sycamore seeds might be beneficial for proper seed germination processes and, putatively, germination speed in this species by modulating the redox status of key actors of this developmental phase. 

The role of NADH- and NADPH-dependent reductases is extremely broad, and their functions are still being elucidated, particularly in seeds [68] because, for now, distinct activities of the two groups of enzymes were reported in dehydrated sycamore seeds and desiccated Norway maple seeds and assigned to contrasting desiccation tolerance of the two *Acer* species [57]. High activity levels were measured for NADH-dependent reductases in sycamore embryonic axes at the three final germination stages (Figure 7). These enzymes may include NADH dehydrogenases used in the electron transport chain for generation of ATP, which peaked at final germination stages at a level twice high compared to the dry stage (Figure 7). NADH for these reactions might originate in sycamore seeds from the activity of GAPDH, which can interact with MsrB1 [92]. Arabidopsis GADPH contains six, nine and nine Met residues in A, B and C2 subunits, respectively. Possibly, GADPH might be kept in active reduced form by MsrB1. Oxidized GAPDH generate more NADPH via pentose phosphate pathway [95]. Thus, we speculate that MsrB1 might be associated with the generation of reducing power. 

The regeneration system of MsrB2 involves a thioredoxin (Trx) and ferredoxin-Trx reductase-dependent mechanism, whereas the mechanism related to MsrB1 uses glutaredoxin (Grx)- or GSH/Grx-dependent mechanisms [52,98]. For MSRB1, the reducing source in seeds could involve NADPH via glutathione reductase and GSH as reported in [98]. For MsrB2, the exact reducing source is very elusive based on what is known in photosynthetic plastids. The activity of NADH-dependent reductases was correlated with the abundance of MsrB1 in sycamore seeds and MsrB2 in Norway maple seeds (Figure 8). Additionally, the abundance of MsrB2 protein was positively correlated with the activity of NADPH-dependent reductases in embryonic axes and cotyledons of Norway maple seeds (Figure 8) suggesting that this group of reductases might be more efficient in Msr regeneration in this species because the activity of NADPH-dependent reductases was higher in this species compared to sycamore (Figure 7). Thus, we suggest based on correlations that NADH or NADPH could be the reducing source for MsrB2 via intermediates that are presently unknown.

## 4. Materials and Methods

### 4.1. Material

All experiments were performed on embryonic axes and cotyledons of seeds belonging to two species of *Acer*, Norway maple and sycamore. Seeds were collected at 23 and 24 weeks after flowering from single trees growing in Kórnik Western Poland, 52°24′37″ N, 17°09′515″ E in the year 2018. Seeds were dried (D) to 10% (Norway maple) and 30% (sycamore) water content. Subsequently, seeds were hydrated for 24 h, and the imbibed seeds (I) were placed in containers on wet towels and kept at 3 °C. Wet towels were changed every week to avoid microorganisms growing. During the germination sensu stricto phase, the material was collected for analyses at regular intervals for each species (for Norway maple every 3 weeks (3, 6, 9), for sycamore every 2 weeks (2, 4, 6, 8)). The last stage collected for analysis constituted germinated seeds (with radicle protruding outside the seed coat) (G). Water uptake was monitored at each analyzed stage via the low-constant-temperature oven method [99]. Moisture content was measured in three lots of 20 embryonic axes and 10 cotyledons each after drying at 17 h at 103 °C and calculated based on fresh weight.

### 4.2. Germination Test

Seeds were imbibed, placed in closed plastic boxes (4, 50 seeds each) and subjected to cold stratification (3 °C) under a 12 h light/12 h dark cycle. Cold stratification of seeds was conducted for 10–12 weeks. Seeds were assayed as germinated when the radicle protruded to 5 mm above the seed testa. Germination speed index (GSI) displaying a time-weighted cumulative germination that measures the speed of germination and quantifies the seedling vigor was calculated according to [100]. 

### 4.3. Determination of ROS Release

#### 4.3.1. H_2_O_2_

The level of H_2_O_2_ release was measured according to the method described by Schopfer et al. [78]. Seeds were incubated in 1.2 mL of a solution containing 20 mM phosphate buffer (pH 6), 5 µM scopoletin and 1 U mL^−1^ peroxidase. The material was incubated in darkness on a shaker at 150 rpm for 1 h at room temperature (RT). After a short centrifugation interval, the fluorescence was measured at an excitation wavelength of 346 nm and an emission wavelength of 455 nm using an Infinite M200 PRO (Tecan, Männedorf, Switzerland) plate reader and Magellan software. The results were shown in picomoles of H_2_O_2_ per gram of dry weight (DW) per hour.

#### 4.3.2. O_2_^•−^

The release of O_2_^•−^ was determined using a method described by Choi et al. [101]. Seeds were incubated in 1.2 mL of solution consisting of 50 mM phosphate buffer (pH 7.8), 0.05% nitro blue tetrazolium (NBT; Sigma, St. Louis, MO, USA) and 10 mM sodium azide. Incubation was performed for 30 min at room temperature in darkness. Subsequently, 750 µL of this reactive solution was heated for 30 min at 85 °C, cooled and centrifuged for 1.5 min at 10,000× *g* RCF. The precipitate was dissolved in dimethyl sulfoxide (DMSO) consisting of 2 M KOH by shaking for 30 min at 150 rpm and vortexing every 5 min. The level of released O_2_^•−^ was measured at 719 nm using an Infinite M200 PRO (Tecan, Männedorf, Switzerland) plate reader and Magellan software. The results are presented as ∆A_719_ values per gram of DW per hour.

#### 4.3.3. •OH

The level of released •OH was determined according to the methods of Schopfer et al. [80]. The material was incubated in 1.2 mL of a reaction mixture containing 20 mM phosphate buffer (pH 6) and 2.5 mM sodium benzoate. Incubation was performed for 3 h at RT in darkness. Moreover, the samples were constituted by shaking at 150 rpm. Then, the solution was briefly centrifuged, and the fluorescence was measured at an excitation wavelength of 305 nm and an emission wavelength of 407 nm using an Infinite M200 PRO (Tecan, Männedorf, Switzerland) plate reader and Magellan software (Tecan, Männedorf, Switzerland). The results were expressed in relative fluorescence units (RFU) per gram of DW per hour. Each analysis was performed on six replicates. For each experiment, six seeds were taken (without separating embryonic axes and cotyledons).

#### 4.3.4. Histochemical Detection of ROS

The method described by Daudi and O’Brien [102] with some modifications mentioned by Kalemba et al. [31] was used to detect H_2_O_2_. Seeds were incubated in a solution of 3,3-diaminobenzidine (DAB) prepared in sodium phosphate buffer. Seeds were incubated in DAB solution for 24 h starting with 30 min of infiltration in the vacuum pump. In the presence of H_2_O_2_, DAB is oxidized and forms an insoluble a reddish-brown polymer. The detection of O_2_^•−^ was performed using a method described by Kumar et al. [103] in which material was incubated for 1 h in 0.2% nitrotetrazolium blue chloride (NBT) in sodium phosphate buffer (pH 7.5). The presence of O_2_^•−^ is visualized as a dark blue color, which is an insoluble formazan dye formed in the presence of a superoxide anion. After incubation, seeds were washed 3 times in water, and subsequently images were taken using a Nikon D3100 digital camera attached to a binocular microscope.

### 4.4. Determination of Peptide-Bound MetO Level

Levels of MetO were determined according to the method of Baxter et al. [104] adapted to seed material [64] using an Agilent Infinity II 1260 model HPLC system (Agilent Technologies, Wilmington, DE, USA) equipped with an Agilent Poroshell 120 Stablebond Aq, 3.0 × 150 mm, 2.7 µm particle column heated to 40 °C, mobile phases based on water (A) and potassium phosphate buffer combined with acetonitrile and isopropanol (B) and the standards of methionine (Met) and MetO with detection wavelengths of 214 nm and a reference at 590 nm. Protein digestion was performed for 20 h at 37 °C with a mixture of proteases including pronase E, leucine aminopeptidase and prolidase. The elution scheme was 0% B from 0.0 to 5.0 min (flow rate of 0.15 mL min^−1^), 0 to 16% B from 5.0 to 8.0 min (flow rate of 0.3 mL min^−1^), 16 to 100% B from 8.0 to 16.0 min (flow rate of 0.3 mL min^−1^) and 0% B from 16.0 to 18.0 min (flow rate from 0.3 to 0.15 mL min^−1^). The MetO ratio was calculated in relation to the total pool of Met detected.

### 4.5. Protein Extraction, Electrophoresis and Western Blot Analysis

Ten embryonic axes and five cotyledons were ground in liquid nitrogen to dry powder, and then the homogenates were incubated in an extraction buffer composed of Tris-Cl, glycerol and β-mercaptoethanol together with polyvinylpolypyrrolidone at 4 °C for 1 h, with shaking every 15 min. The homogenates were centrifuged for 20 min at 20,000× *g* at 4 °C. The protein concentration was measured using the Bradford [105] method. Proteins were separated by SDS-PAGE on 12% polyacrylamide gels, with an equal amount of protein (20 μg) in each lane (Figure S1). The Western blot analysis was performed according to the method described by Wojciechowska et al. [64]. The primary antibodies anti-MsrB1 and anti-MsrB2 [87] were diluted 1:1000 in 5% skimmed milk/PBS. Secondary antibodies conjugated with horseradish peroxidase (HRP, catalog number AS09 602, Agrisera, Sweden) were diluted 1:10,000 in 5% skimmed milk/PBS. Western blot results were analyzed densitometrically in triplicate using the UviBand (UviTec, Cambridge, UK) program of the Fire Reader Gel Documentation System. The density of the band was calculated based on the volume (V) of the band as the sum of all 3D intensities (I) coded on a scale of 256 gray levels. The data show relative units obtained from V = Σn_i_I and the number of pixels inside the area of the band.

### 4.6. Activity of NAD(P)H-Dependent Enzymes

Activity of NAD(P)H-dependent enzymes was measured according to method describing by Alipour et al. [57] based on the reduction of 5,5′-dithiobis(2-nitrobenzoic) acid (DTNB) with NAD(P)H to 2-nitro-5-thiobenzoate. The results of the reaction were measured using an Infinite M200 PRO (TECAN) plate reader and Magellan software.

### 4.7. Statistical Analyses

All experiments were performed with three independent biological replicates. Statistically significant differences were indicated with different letters (ANOVA and Tukey’s test at *p* > 0.05). Pearson’s correlation coefficient analysis was used to evaluate the relationship between particular parameters. Proportional data were transformed prior to analysis using the arcsine transformation. R statistical software was used to calculate Pearson’s correlation coefficients [106]. The corrplot package was used to construct correlation matrices [107].

## 5. Conclusions

Seed germination has never been investigated in relation to Met redox homeostasis. Norway maple and sycamore seeds representing orthodox and recalcitrant categories, respectively, were found to display distinct behavior, sycamore exhibiting a higher germination speed. Both were highly viable, but also differed in ROS and MetO levels, MsrB abundance and global reducing power. ROS peaks, especially those coinciding with MetO peaks, were assigned as ROS-related events linked to faster germination of sycamore seeds. In sycamore, the negative correlation between MetO and MsrB2 indicates that the reversible MetO posttranslational modification could contribute to the fast germination observed in this species. We suggest that balanced Met redox status is an important feature of sycamore seeds contributing to their high germination speed. Further identification of MsrB targets in germinating seeds would help to decipher their roles in repair mechanisms or signaling pathways during this key developmental stage.

## Supplementary Materials

Supplementary Materials can be found at https://www.mdpi.com/1422-0067/21/23/9197/s1.

## Abbreviations

•OH	Hydroxyl radicals
ABA	Abscisic acid
Cys	Cysteine
DAB	3,3-diaminobenzidine
DW	Dry weight
Gas	Gibberellins
GAPDH	Glyceraldehyde-3-phosphate dehydrogenase
Grx	Glutaredoxin
GSI	Germination speed index
H_2_O_2_	Hydrogen peroxide
HRP	Horseradish peroxidase
Met	Methionine
MetO	Methionine sulfoxide
Msrs	Methionine sulfoxide reductases
NADPH	Nicotinamide adenine dinucleotide phosphate
O_2_^●−^	Superoxide anion radicals
NBT	Nitrotetrazolium blue chloride
PPP	Pentose phosphate pathway
RFU	Relative fluorescence units
ROS	Reactive oxygen species
RT	Room temperature
Trx	Thioredoxin
WAI	Week after imbibition
