# Supplementary material for: Involvement of the MetO/Msr System in Two Acer Species That Display Contrasting Characteristics during Germination
Source: Int J Mol Sci. 2020 Dec 2;21(23):9197. doi: 10.3390/ijms21239197 (PMC7730483; doi:10.3390/ijms21239197)

Figure S1

SDS-PAGE representative gels of protein extracts (20 µg) isolated form embryonic axes and cotyledons of Norway maple (A) and sycamore (B) seeds further used for Western blot analyses. Analyses concerned proteins originating from D-dry seeds, I - imbibed seeds, stratified seeds collected at 2- and 3-weeks intervals and G- germinated seeds with visible elongated radicle. The Spectra^{TM} Multicolor Broad Range Protein Ladder marher (Thermo Scientific) was used to calculate the molecular weight. Gels were documented with using G:BOX Chemi XR5 instrument (Syngene, Cambridge, UK) and Coomassie Blue filter settings.

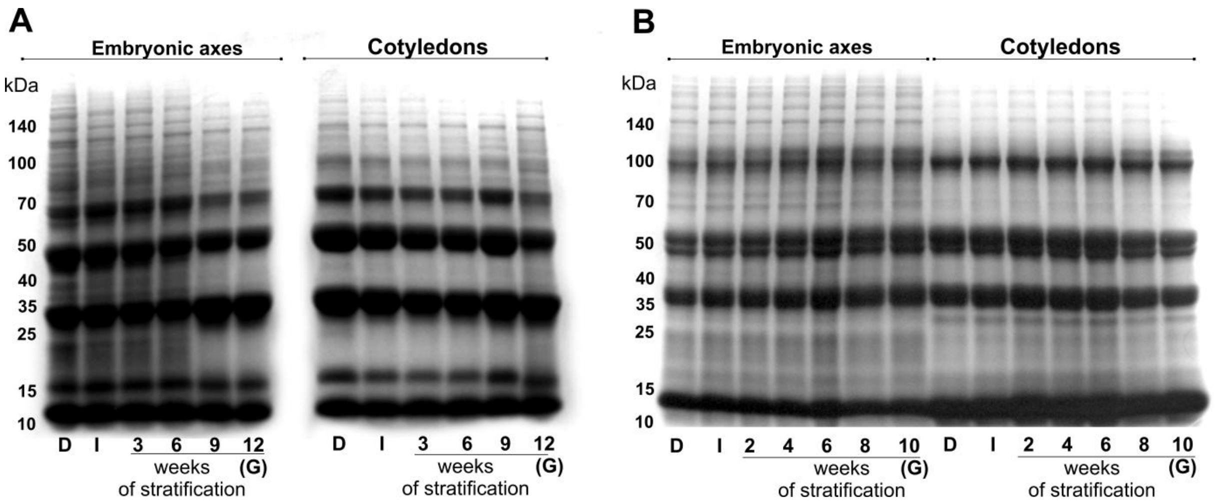

Supplement: Supplementary file 1 [file ijms-21-09197-s001.pdf]
